# Natural variation in the chickpea metabolome under drought stress

**DOI:** 10.1111/pbi.14447

**Published:** 2024-10-16

**Authors:** Palak Chaturvedi, Iro Pierides, Cristina López‐Hidalgo, Vanika Garg, Shuang Zhang, Rutwik Barmukh, Anke Bellaire, Jiahang Li, Gert Bachmann, Luis Valledor, Rajeev K. Varshney, Arindam Ghatak, Wolfram Weckwerth

**Affiliations:** ^1^ Molecular Systems Biology Lab (MOSYS), Department of Functional and Evolutionary Ecology University of Vienna Vienna Austria; ^2^ Plant Physiology, Department of Organisms and Systems Biology Faculty of Biology, and Biotechnology Institute of Asturias, University of Oviedo Oviedo Spain; ^3^ WA State Agricultural Biotechnology Centre, Centre for Crop & Food Innovation Food Futures Institute, Murdoch University Murdoch WA Australia; ^4^ Structural and Functional Botany, Department of Botany and Biodiversity Research University of Vienna Vienna Austria; ^5^ Vienna Metabolomics Center (VIME) University of Vienna Vienna Austria

**Keywords:** *Cicer arietinum*, metabolomics, stress susceptibility index (SSI), metabolic GWAS, data‐driven control analysis, Lyapunov equation

## Abstract

Chickpea is the world's fourth largest grown legume crop, which significantly contributes to food security by providing calories and dietary protein globally. However, the increased frequency of drought stress has significantly reduced chickpea production in recent years. Here, we have performed a field experiment with 36 diverse chickpea genotypes to evaluate grain yield, photosynthetic activities and molecular traits related to drought stress. For metabolomics analysis, leaf tissue was collected at three time points representing different pod‐filling stages. We identified L‐threonic acid, fructose and sugar alcohols involved in chickpea adaptive drought response within the mid‐pod‐filling stage. A stress susceptibility index for each genotype was calculated to identify tolerance capacity under drought, distributing the 36 genotypes into four categories from best to worst performance. To understand how biochemical mechanisms control different traits for genetic improvement, we performed a differential Jacobian analysis, which unveiled the interplay between various metabolic pathways across three time points, including higher flux towards inositol interconversions, glycolysis for high‐performing genotypes, fumarate to malate conversion, and carbon and nitrogen metabolism perturbations. Metabolic GWAS (mGWAS) analysis uncovered gene candidates involved in glycolysis and MEP pathway corroborating with the differential biochemical Jacobian results. Accordingly, this proposed data analysis strategy bridges the gap from pure statistical association to causal biochemical relations by exploiting natural variation. Our study offers new perspectives on the genetic and metabolic understanding of drought tolerance‐associated diversity in the chickpea metabolome and led to the identification of metabolic control points that can be also tested in other legume crops.

## Introduction

The global human population is experiencing exponential growth, while the agricultural sector is not expanding at the same rate. There is a pressing need to boost crop production to address the increasing dietary demands. However, this endeavour is hindered by challenges such as heatwaves, droughts, and other unpredictable environmental conditions. The ‘climate crisis’ leads us towards a warmer and drier Earth (FAO, 2019). Approximately US $29 billion in global economic losses in agriculture stemming from drought in the last decade were reported (FAO, 2018). Water demand for agriculture by 2050 could increase twofold, with freshwater availability decreasing by up to 50% due to increasing climatic variations. To achieve food security, there is an urgent need to revamp investments in developing high‐yielding crops that are climate resilient and more efficient in up‐taking water than their existing counterparts (Atlin *et al*., 2017; Trnka *et al*., 2019; Varshney *et al*., 2021a).

Chickpea (*Cicer arietinum* L.) is one of the major grain legumes, with a global annual production of ~15.87 million tonnes from an area of ~15.00 million hectares. It possesses an average yield of 1.06 tonnes/hectares (FAOSTAT, 2021). Chickpea contributes significantly to the world's food security by providing dietary proteins and calories for millions of people (Varshney *et al*., 2013). Rainfed conditions contribute almost 80% of chickpea production in the fields (Khan *et al*., 2019; Pang *et al*., 2017). However, the production of chickpea in rainfed systems faces significant limitations, primarily due to drought stress in the latter stages of growth (known as terminal drought), which leads to substantial yield losses, averaging around 64% in India (Hajjarpoor *et al*., 2018) and approximately 40–90% globally depending upon the timing and severity of water stress (Fang *et al*., 2010; Korbu *et al*., 2022; Leport *et al*., 2006; Nayyar *et al*., 2006). Chickpea seed yield decreases significantly during terminal drought due to reduced pod production, seed size, and flower and pod abortion (Leport *et al*., 1999, 2006). Developing chickpea varieties that are more productive under occasional drought scenarios has been successful over the past few decades through conventional breeding (Hajjarpoor *et al*., 2018; Kashiwagi *et al*., 2006). However, these approaches alone are insufficient to keep pace with the future food demand. To this end, rapid identification of genetic variation underlying crop performance can improve breeding efficiency and substantially accelerate resilient crop improvement (Roorkiwal *et al*., 2020; Varshney *et al*., 2021b). For example, chickpea varieties have been characterized for quantitative trait loci associated with drought tolerance‐related traits, including root architecture, transpiration efficiency and early vigour (Barmukh *et al*., 2022). However, it is also extremely important to understand biochemical mechanisms controlling different traits for genetic improvement. Hence, analyses of biologically important molecules are necessary to understand stress response mechanisms in chickpea.

Plants release numerous biochemical compounds, including major metabolites, under abiotic stress conditions (Ghatak *et al*., 2018). Plant metabolites (primary and secondary) play essential roles in cell signalling, membrane formation, cell integrity, energy storage, growth, plant development, cellular replenishment and whole‐plant resource allocation (Ghatak *et al*., 2018). Plants adapt to different conditions (biotic and abiotic) through metabolic changes and modify their physiology accordingly (Chaturvedi *et al*., 2024; Weckwerth *et al*., 2020). An untargeted metabolomics study in chickpea under stress revealed 20 known metabolites, of which proline, arginine, histidine and tryptophan were increased, and aspartic acid, alanine, tyrosine and phenylalanine decreased under drought stress (Khan *et al*., 2019). Kudapa and co‐workers recently employed a multi‐omics approach to study chickpea roots under drought stress considering four genotypes with contrasting responses to drought stress, viz., ICC 4958 (drought‐tolerant), JG 11 (drought‐tolerant), an introgression line JG 11+ (drought‐tolerant) and ICC 1882 (drought‐sensitive). This investigation identified six metabolites (fructose, galactose, glucose, myo‐inositol, galactinol and raffinose) that significantly correlate with RFO metabolism (Kudapa *et al*., 2024). In yet another study, different genotypes of chickpea were evaluated under rainfed and irrigated field conditions, which revealed significant differences in several metabolites, including oxalic acid, threonic acid, inositol, maltose and L‐proline between studied groups (Nisa *et al*., 2020). Chickpea subjected to drought stress uncovered co‐expressed genes, proteins and metabolites regulating glutathione metabolism, glycolysis/gluconeogenesis and phosphatidylinositol signalling pathways. Significant alterations were observed in the drought‐tolerant genotype (Singh *et al*., 2023).

During drought stress, plants decrease stomatal conductance, resulting in reduced CO_2_ fixation and a decrease in the rate of photosynthesis, followed by a reduction in growth and seed yield (Pang *et al*., 2017). Plants can protect themselves against mild drought stress in an emergency by accumulating osmolytes (Todaka *et al*., 2017). Osmolytes are small organic compounds that serve as compatible solutes in plants, as they are non‐toxic to plant cells and do not disrupt regular metabolic processes. The key inquiry in comprehending legume stress revolves around investigating osmolytes and other small molecules to observe how plants respond to stress and adapt to uphold their internal balance or homeostasis. Metabolomics is one key technique to achieve this goal, which plays an important role in understanding the complex shifts that occur in plants under environmental perturbations, such as drought and limited water stress (Feussner and Polle, 2015).

One of the main challenges for plant breeders is selecting genotypes that could handle environmental stress like drought. Several selection indices have been suggested to differentiate the degree of stress resistance between different genotypes (Ayed *et al*., 2021). One important index is the stress susceptibility index (SSI), which was first employed by Fischer and Maurer (Fischer and Maurer, 1978). This index describes the variation of yield performance under stress and non‐stress conditions, allowing the breeders to exploit genetic variation to screen stress‐tolerant varieties. SSI was employed in several studies, for example, the evaluation of drought tolerance in durum wheat genotypes, which revealed year‐to‐year and location‐to‐location variation (Mohammadi *et al*., 2011). Similarly, it was applied to identify susceptible cotton genotypes under rainfed conditions (Nandhini *et al*., 2022). Recently, Nouraei and co‐workers identified 53 single‐nucleotide polymorphisms (SNPs) significantly associated with SSI in a wheat genome‐wide association study of drought tolerance (Nouraei *et al*., 2024). The response mechanism under drought stress varies with the genotypes and developmental stage of the plants. Hence, it would be much more valuable if biochemical indicators could be identified for each crop species. Additionally, interrelationships among various physiological responses to dehydration can provide insight into developing useful strategies to improve drought stress response in chickpea. Therefore, the objectives of the present study were: (1) to examine the impact of drought stress on leaf metabolism at three pod‐filling stages in a natural field environment, using Gas chromatography–mass spectrometry (GC–MS) analysis; (2) to gain insights into the intricate physiological and molecular changes that occur in response to stress conditions, their impact on yield, and to assess the SSI among different chickpea genotypes with a focus on Desi varieties to provide a homogenous population; and (3) to employ bio‐mathematical methods to understand the plasticity between molecular and phenotypic networks, which are the main facilitators of adaptive changes in response to stressful environments. The exploration of constraints that facilitate plasticity in metabolic networks were determined using a data‐driven approach in chickpea. In this study, we exemplify the use of the differential biochemical Jacobian matrix (Doerfler *et al*., 2013; Kitashova *et al*., 2023; Li *et al*., 2023; Nagele *et al*., 2014; Nukarinen *et al*., 2016; Sun and Weckwerth, 2012; Weckwerth, 2019; Weiszmann *et al*., 2023; Wilson *et al*., 2020) to decipher metabolic constraints of the adaptive response to drought stress and its association to SSI as well as metabolic genome‐wide association study (mGWAS) in chickpea. Additionally, we highlight its adaptability in metabolomics studies to explore valuable traits that could be integrated into the breeding programs. (4) Finally, a mGWAS analysis was conducted for identifying the genomic regions involved in metabolite alterations between well‐watered and drought conditions as well as investigating a relation between SNP associations with differential metabolic fluxes derived from the differential biochemical Jacobian (Weiszmann *et al*., 2023).

## Results

### Experimental setup and physiological measurements

**Figure 1:**
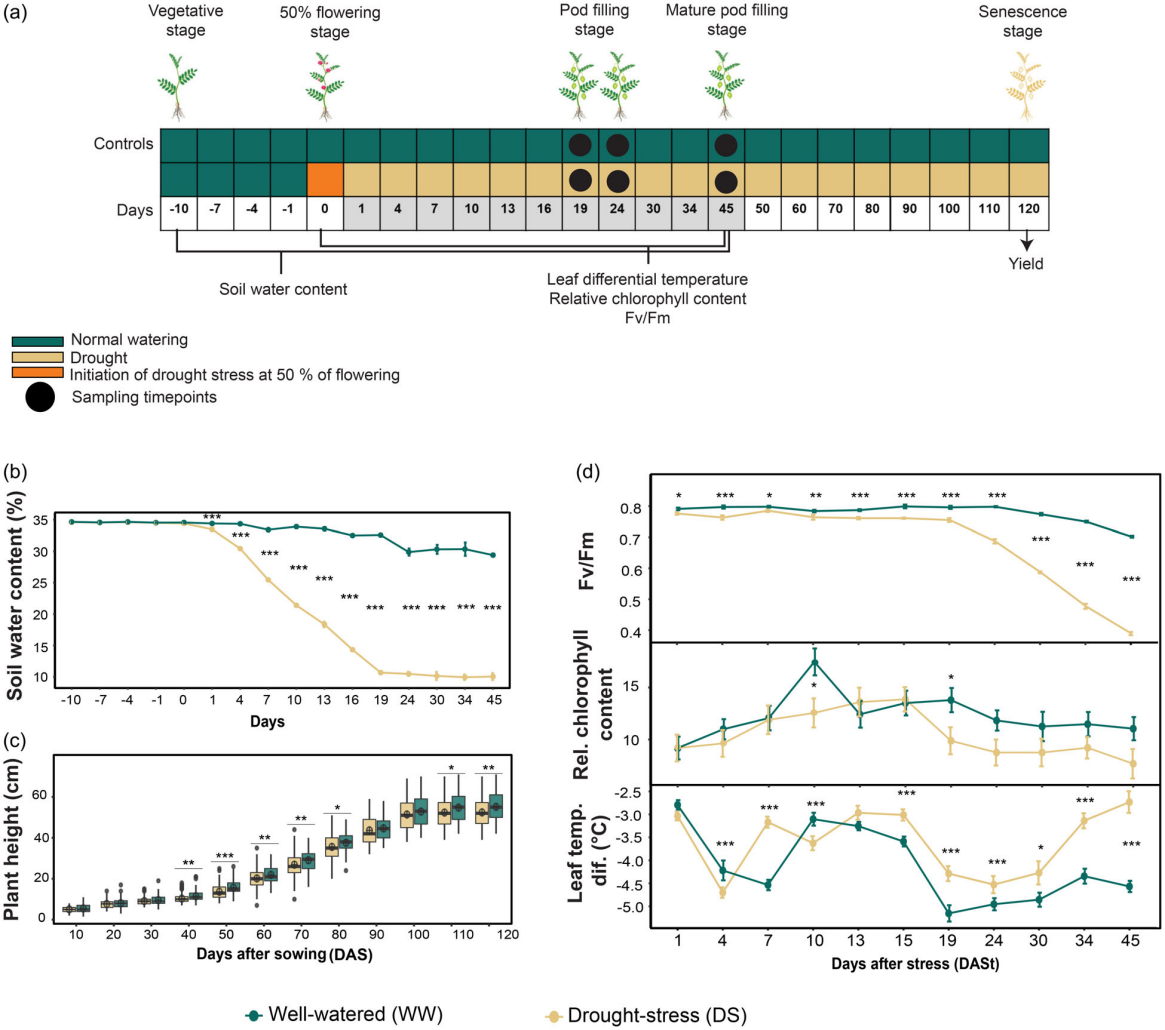
(a) Experimental design. (b) Soil water content (SWC) (%). Values represent means, and error bars indicate the corresponding standard errors (*n* = 6). (c) Plant height (in cm). Boxplots representing the plant height measured. The boxplots show the median (central bar), the mean (central circle), the interquartile range (box), and minimum and maximum values (vertical bars). The circles outside the box show the outliers. The colour of the boxplots represents the different experimental conditions. (d) Maximum photochemical efficiency of PSII (*F*_*v*_/*F*_*m*_), relative chlorophyll content and leaf temperature differential. Values represent mean, and error bars indicate the corresponding standard errors (*n* = 108). Means without at least one asterisk are non‐significant from each other (*P* > 0.05; one‐way ANOVA) between the treatments well‐watered (WW) and drought‐stressed (DS). Significance was tested for each time. Significance per day is indicated by asterisk (***P < 0.001; **P < 0.01; *P < 0.05).

In the present study, 36 diverse chickpea genotypes were evaluated under drought stress (DS) in the natural field condition. Drought stress was initiated when plants were at 50% flowering stage (Figure 1a). The significant difference in the soil water content (%) between well‐watered (WW) and DS conditions was the first indication of the drought stress imposed. Initial significant change in soil water content (%) was observed on 7 days after stress (DASt), which plateaued after 19 DASt (Figure 1b). The measurement of plant height (in cm) was taken throughout the developmental stages, from the vegetative phase to maturity. A significant decrease in plant height was noted during the flowering and pod‐filling stages under DS conditions (Figure 1c). To investigate the physiological basis of genotypic variation under drought stress, photosynthetic parameters were determined using PhotosynQ V2.0, which include leaf temperature differential (corresponding to the stomatal closure), *F*_*v*_/*F*_*m*_, and relative chlorophyll content (see section ‘Materials and Methods’). The recorded observations were analysed in parallel to define the harvesting time points for further molecular analysis. All the recorded observations are provided in Table S1.

The immediate response of plants under DS is the closure of stomata to prevent water loss via transpiration. Plants grown under drought conditions tend to have lower stomatal conductance, thus helping to conserve water and maintain an adequate leaf water status while reducing leaf internal CO_2_ concentration and photosynthesis (Ghatak *et al*., 2020). The precise relationship also depends on factors like genotypes, drought history and environmental conditions. In this study, leaf temperature differential showed a significant difference under DS compared to WW on 19 DASt, followed by a slight increase on 24 DASt, with a substantial difference between DS and WW on 45 DASt (Figure 1d). In general, the relative chlorophyll content decreased in all the genotypes under DS compared to the WW condition, with a significant decrease observed at 19 DASt (Figure 1d). All the genotypes studied showed a significant decrease in the photochemical efficiency of photosystem II (*F*_*v*_/*F*_*m*_) at different time points after the initiation of DS (Figure 1d). Considering the physiological response, the different harvesting time points were selected, which corresponded well with the pod‐filling stage in chickpea. The first harvesting point was selected at the early pod‐filling stage (19 DASt), the second was at the mid pod‐filling stage (24 DASt), and the third sampling point was at the late pod‐filling stage (45 DASt) (harvest time point 3) (Figure 1a). The plants were allowed to grow until the senescence stage to evaluate the impact of drought stress on yield, which also helped to categorize the genotypes based on their degree of drought tolerance (see section on SSI).

### Stress susceptibility index (SSI) and its correlation with differential metabolomic response under drought stress in chickpea

**Figure 2:**
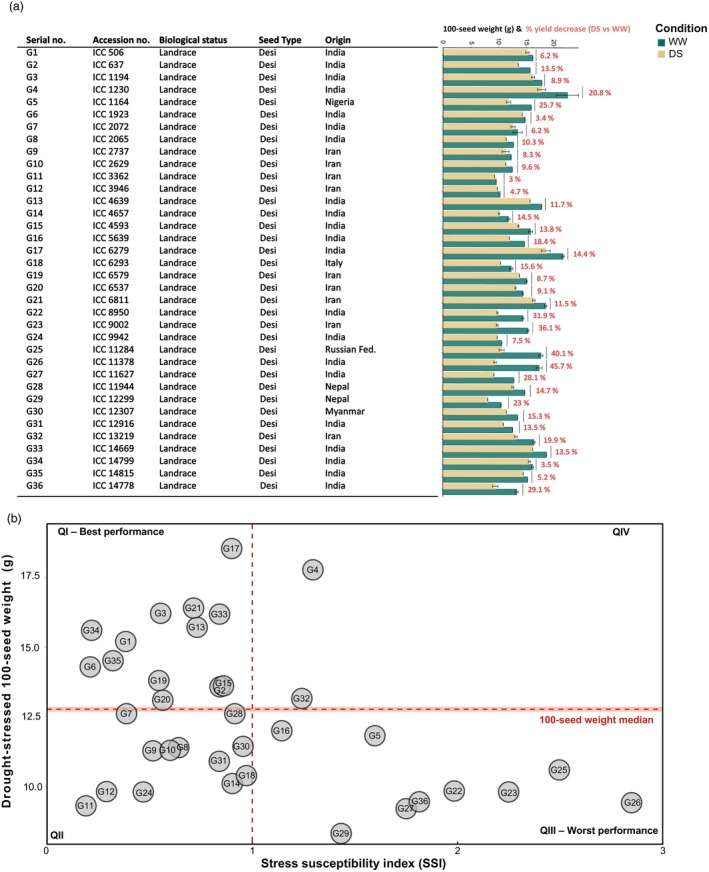
(a) Natural variation in 100‐seed weight among chickpea genotypes with 100‐seed weight percentage decrease under drought stress. (b) Biplot with stress susceptibility index (SSI) and drought‐stressed 100‐seed weight of each chickpea genotype.

The list of genotypes and their percentage yield reduction due to DS is indicated in Figure 2a. To differentiate the degree of drought tolerance and evaluate the grain yield potential under DS among different chickpea genotypes, we determined the SSI, a ratio of genotypic performance under stress and non‐stress conditions (Talebi *et al*., 2013) (Table S1). Figure 2b shows the biplot between maximum production under DS conditions (100‐seed weight under DS samples) and SSI for 36 chickpea genotypes (Gutiérrez *et al*., 2020). In the biplot, quadrant I (Q1) corresponds to chickpea genotypes that are drought tolerant and have a high production capacity under stress conditions (designated as best chickpea genotypes from the current germplasm), quadrant II (Q2) contains chickpea genotypes that are tolerant to DS while having low 100‐seed weight under stress conditions, quadrant III (Q3) corresponds to susceptible chickpea genotypes with low 100‐seed weight, and quadrant IV (Q4) contains chickpea genotypes susceptible to DS with high production under drought conditions (Figure 2b).

With the help of SSI, we were able to categorize tolerant genotypes under DS conditions. Values less than 1 for SSI indicate genotypes with more tolerance to water stress, while those with values over 1 are more susceptible. The SSI values for 100‐seed weight ranged from 0.21 to 2.85 (Table S1). Thus, the SSI values between 0.21 and 0.97 of several chickpea genotypes (QI and II) indicated that these genotypes could be considered more tolerant to DS. The chickpea genotypes located in QI were G6, G34, G35, G1, G19, G20, G3, G21, G13, G33, G17, G15 and G2. These genotypes can be considered best performing ones due to the highest 100‐seed weight value under DS and the lowest value of SSI. However, chickpea genotypes with higher SSI values (1.14 – 2.85) can be considered to have lower drought tolerance (QIII and IV). The chickpea genotypes located in QIII were G26, G25, G23, G22, G36, G27, G29, G5 and G16 (Figure 2b). These genotypes can be considered the worst performing ones due to the lowest 100‐seed weight value under DS and the highest value of SSI. Moreover, this drought index‐based genotypic ranking consistently agrees with the results of chickpea genotype responses under DS expressed as percentage yield reduction (Figure 2a).

**Figure 3:**
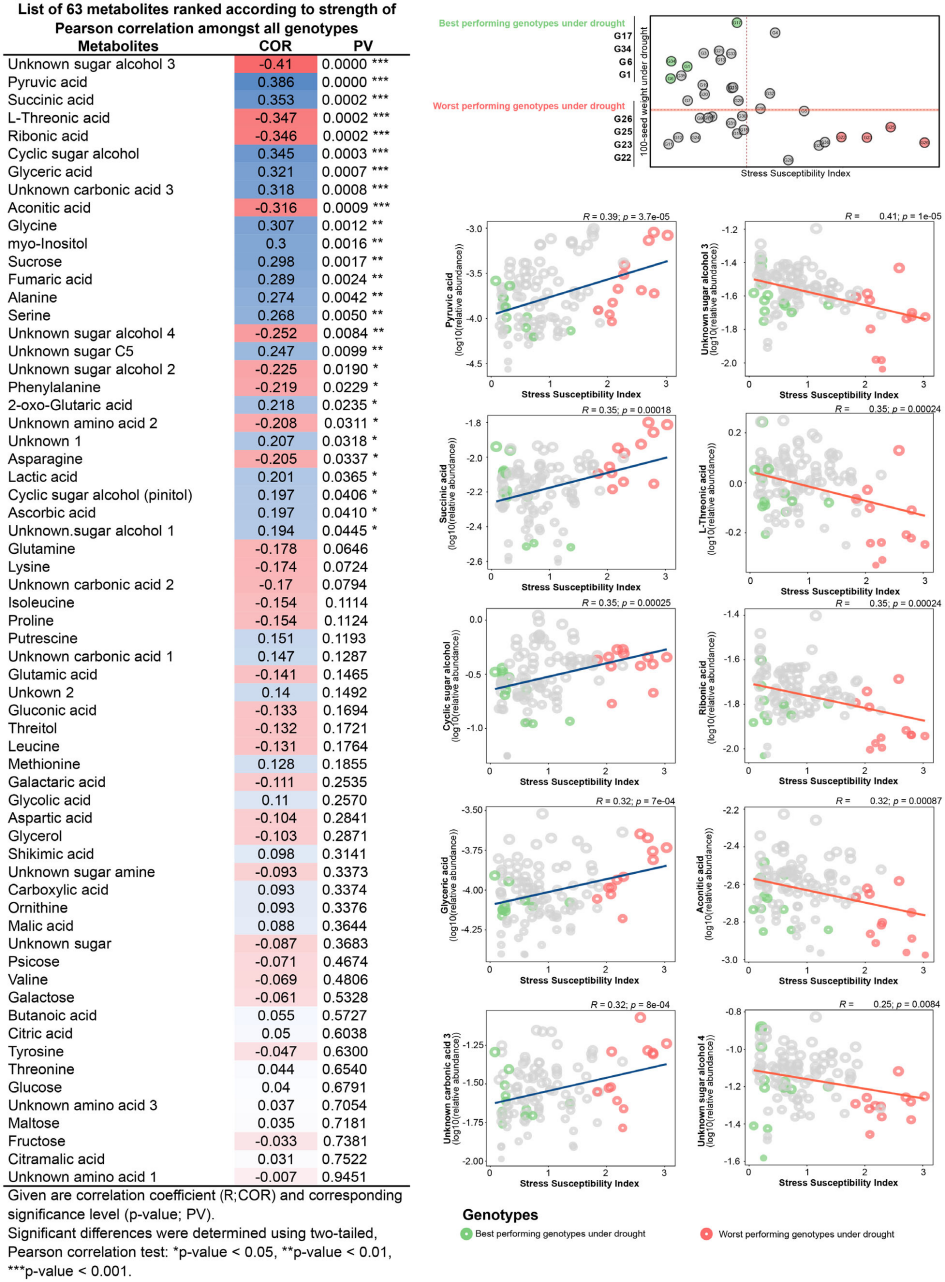
Pearson correlation analysis to distribute the 36 diverse chickpea genotypes based on the performance concerning the metabolite and SSI (Stress Susceptibility Index) value. The pairwise correlations between all 63 measured metabolite levels and SSI were calculated. Positive correlations are shown in blue; negative correlations are shown in red. The intensity of blue or red represents the value of the correlation coefficient. The * indicates a statistically highly significant correlation (*P*‐value < 0.05) (Left panel). Metabolite‐SSI biplot shows the correlation strength between metabolites and SSI (Right panel). The size of each circle denotes the relative amount value of metabolites with the corresponding chickpea genotype. Genotypes with a lower SSI value and a higher 100‐seed weight value have been considered the best genotypes. Genotypes with a higher SSI value and a lower 100‐seed weight value have been considered the worst genotypes.

Once each chickpea genotype's SSI was evaluated, we examined the prediction power of metabolites to prove the hypothesis that metabolome can be related to the SSI and, consequently, seed quality and production. For that, the distribution of single metabolites from mid‐pod‐filling stage (i.e. harvest time point 2) was queried for their predictive power concerning the SSI distribution by calculating pairwise correlations between all 63 measured metabolites relative abundance and 36 chickpea genotypes with SSI values (Figure 3). The 63 metabolites are listed in Figure 3 (*left panel table*) (harvest time point 2). Metabolites with the highest significant correlations are displayed on the two‐dimension correlation plot (log‐transformation of metabolite relative abundance and SSI values) (Figure 3 (*right panel*)), which strongly represents central metabolism‐derived compounds such as organic acids (pyruvic acid, succinic acid, glyceric acid, unknown carbonic acid 3, L‐Threonine acid, ribonic acid and aconitic acid) and sugar alcohols (cyclic sugar alcohol, unknown sugar alcohol 3 and unknown sugar alcohol 4). Some metabolites are tricarboxylic acid (TCA) cycle members, such as succinic acid, aconitic acid and pyruvic acid. The highest absolute correlation (COR, R) found was for sugar alcohol compounds, which yielded a value of −0.41. This correlation is statistically significant (*P*‐value of 1.036 × 10^−5^) and can explain 16.08% of the variance. Other significantly correlated compounds include pyruvic acid (0.386; *P*‐value of 2.709 × 10^−5^), succinic acid (0.353; *P*‐value of 0.0002), L‐Threonic acid (−0.347, *P*‐value of 0.0002), ribonic acid (−0.346; *P*‐value of 0.0002) and cyclic sugar alcohol (0.345; *P*‐value of 0.0003).

### Metabolome profiling

**Figure 4:**
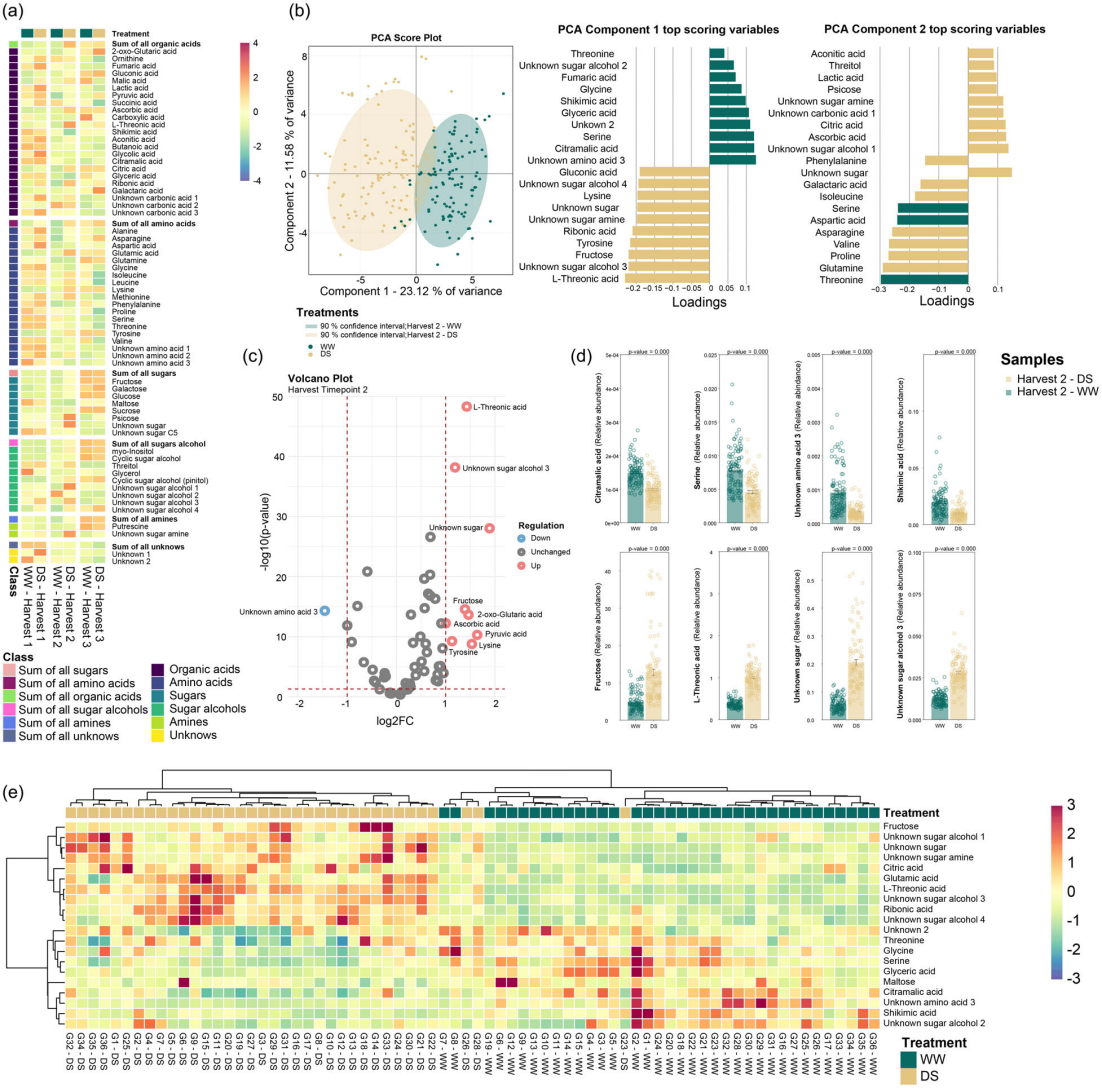
(a) Heatmap ‐ Metabolic changes in leaves under drought stress (left to right) harvest time point 1 (19 DASt), harvest time point 2 (24 DASt), harvest time point 3 (45 DASt). Metabolic changes are presented as means of each treatment. Colours indicate increases (red) and decreases (blue). (b) PCA Score plot and PC1 and PC2 top‐ranked metabolites in harvest time point 2. The top 20 scoring loadings (10 highest and 10 lowest) of PC1 and PC2 are shown by row for each PCA. Bar colours indicate the experimental condition in which each top‐scoring metabolite is more regulated. Ellipses show a 90% confidence interval. Different colours indicate different experimental conditions (*n* = 108 biologically independent replicates). (c) Volcano plot generated with a 216‐observation dataset (108 well‐watered (WW) samples and 108 drought‐stressed (DS) samples) comprised of 63 metabolites at harvest time point 2. Red circles represent metabolites with a fold change ≥2 that was statistically significant (*P*‐value ≤ 0.05). Blue circles represent metabolites with a fold change ≥2 lacking statistical significance (*P*‐value > 0.05), while grey circles represent metabolites with a fold change ≤2. The identity of the most discriminating metabolites highlighted in the plot as red and blue circles are described in bar plots. (d) Barplot visualization of the highlighted metabolites in WW and DS conditions. Error bars represent the standard error on the 108 replicates. (e) Hierarchically clustered heatmap of the 36‐chickpea genotypes using the top 20 metabolites with higher loadings in the first component of PLS‐DA. The bi‐clustering uses the average linkage of Pearson correlation distance between chickpea genotypes and metabolites. Metabolic changes are presented as means of 3 replicates. Colours indicate increases (red) and decreases (blue).

Metabolome profiling of the leaf tissue was conducted using GC‐time‐of‐flight (TOF)‐MS. Sixty three metabolites were identified and quantified with identification level 1 using quality control (QC) mixes and in‐house library (Ghatak *et al*., 2022; Weiszmann *et al*., 2023; Zhang *et al*., 2021). The identified metabolites were classified into organic acids, amino acids, sugars, sugar alcohols, amines and unknowns represented using the heat map (Figure 4a; Table S2). Principle component analysis (PCA) revealed distinct metabolites associated with DS and WW samples at three different time points (Figure S1; Table S3), suggesting a clear distinction in the metabolite accumulation under two conditions. Harvest time point 2 demonstrated the strongest separation between DS and WW conditions at PC1 compared to harvest time points 1 and 3 (Figure 4b). The highest positive loading of PC1 indicates metabolites that were highly accumulated in the WW condition compared to DS, which includes threonine, unknown sugar alcohol 2, fumaric acid, glycine, shikimic acid, serine, etc. The highest negative loadings of PC 1 indicate that metabolites highly accumulated under DS were unknown sugar alcohol 3, L‐threonic acid, fructose, tyrosine and unknown sugar, among others (Figure 4b). Further, PLS‐DA analysis was performed for DS and WW conditions, which also demonstrated a similar outcome with the strongest variation at harvest time point 2 (with PC1 explaining 21.64% total variation) compared to harvest time points 1 and 3 (Figure S2; Table S4).

Since harvest time point 2 (i.e. mid pod‐filling stage) was the most drought‐responsive time point, we focused on evaluating the metabolome identified at this stage. Metabolites with significant accumulation (*P* value <0.05) under DS determined by a volcano plot (Figure 4c) include L‐threonic acid, unknown sugar alcohol 3, pyruvic acid, ascorbic acid, fructose, and 2‐oxo‐glutaric acid. The bar plot represents the regulation of these selected metabolites across all the replicates under DS and WW conditions (Figure 4d). Regulation of all 63 metabolites across all the replicates in both DS and WW conditions can be found in Figure S3. Based on the outcome of PLS‐DA analysis (Table S4), heat maps were constructed to determine the regulation of the metabolites at harvest time point 2 (Figure 4e) and similarly for harvest time points 1 and 3 considering all the genotypes (Figure S4).

Metabolite clustering was performed using K‐means clustering across all three time points. In total, *K* = 10 clusters were identified based on the accumulation of patterns of the metabolites in three harvesting time points (Figure S5; Table S5). It indicated metabolic changes under DS across three pod‐filling stages.

### Biochemical Jacobian analysis for drought stress tolerance in chickpea

To complement and enhance the conclusions drawn from the statistical analysis, we include the Jacobian matrix as a dynamic systems tool that can act as a bridge between statistics and mathematical formalisms of metabolic networks (Weckwerth, 2019). While purely statistical methods such as PCA, PLS‐DA and clustering approaches offer great insight into distributions and correlations in the data, dynamic system tools consider non‐static interactions and aim to decipher mechanisms that might explain the measured observables in the system. The Jacobian matrix is a linearization of a system's steady state (Tailor and Bhathawala, 2011) and approximates a system's dynamics around a specific time point, omitting complex non‐linearities. It provides the rate of change of the system and indicates interrelations among variables, represented as partial derivatives as in:

(1) $$J=\left[\begin{array}{cccc} \frac{\partial f1}{\partial x1} & \frac{\partial f1}{\partial x2} & \ldots & \frac{\partial f1}{\partial \mathrm{xn}}\\ \frac{\partial f2}{\partial x1} & \frac{\partial f2}{\partial x2} & \ldots & \frac{\partial f2}{\partial \mathrm{xn}}\\ \vdots & \vdots & \ddots & \vdots \\ \frac{\partial \mathrm{fn}}{\partial x1} & \frac{\partial \mathrm{fn}}{\partial x2} & \ldots & \frac{\partial \mathrm{fn}}{\partial \mathrm{xn}} \end{array}\right]$$

Each entry gives information on how fast or slow a reaction changes its rate according to the rate of change of a metabolite's concentration, i.e. its sensitivity towards that metabolite, known as ‘reaction elasticity’ (Heinrich and Schuster, 1996). Larger entry values indicate a faster rate of change with respect to a metabolite's concentration and smaller values indicate slower rates of change (Table S6).

**Figure 5:**
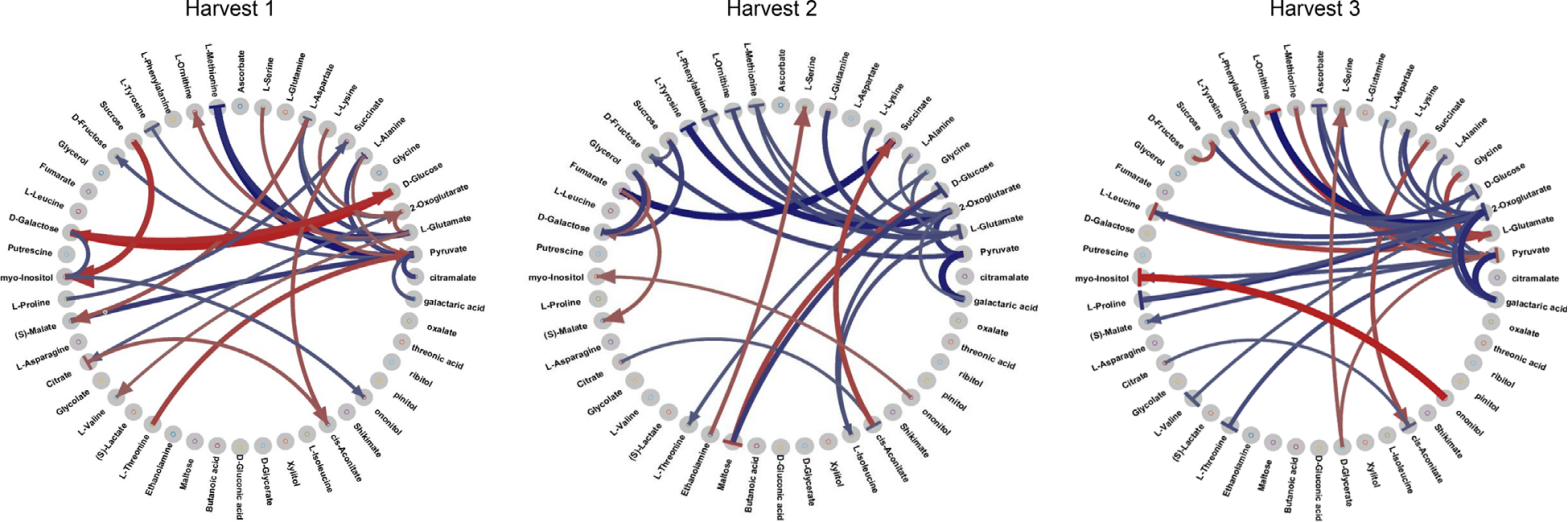
Circular plot for the differential Jacobian entries between Q1 and Q3 chickpea genotypes within three harvest time points under DS. Red/blue colours indicate higher reaction rates for Q1/Q3 genotypes respectively. The shape of the arrow indicates whether this reaction is activating (→) or inhibiting (‐‐|).

Here we make use of the differential Jacobian approach (Nägele *et al*., 2014; Nagler *et al*., 2018; Weiszmann *et al*., 2023) to compare high‐performing (Q1) versus low‐performing (Q3) chickpea genotypes and to identify which metabolic reactions contribute to a high yield index and drought tolerance. Focusing on the primary drought response within harvest time point 2, differential reaction elasticities are indicated in Figure 5, with red/blue arrows corresponding to larger differential entries in Q1/Q3 genotypes respectively. The arrow shape indicates whether a reaction is activated or inhibited. The reaction elasticities fumarate to malate, cis‐aconitase to succinate, ethanolamine to serine, and ononitol to myo‐inositol are shown to have larger, activating differential values in Q1 compared to Q3 chickpea genotypes. Conversely, reaction elasticities from maltose to glucose and those leading to tyrosine, phenylalanine, methionine, and ornithine have relatively larger inhibiting fluxes in Q3 genotypes. Differential metabolite fluxes for all the three time points are shown in Figure 5 and further discussed in Document S1.

### Metabolic genome‐wide association study reveals genomic regions associated with drought tolerance in the chickpea metabolome

Next, to identify the genomic regions associated with drought tolerance in the chickpea metabolome, we performed mGWAS using the available genotypic data (Varshney *et al*., 2021b). In total, we obtained 334 significant marker‐trait associations (MTAs) from covering 292 SNPs across different combinations of harvest time points and treatment (Table S7). The significant MTAs were found for metabolites such as galactaric acid (SNP: S5_10723119) and unknown sugar amine (SNPs: S4_48687551 and S1_13338812) for harvest time point 1, asparagine (SNP: S3_37239676) and unknown sugar alcohol 4 (SNP S5_40024599) for harvest time point 2 and succinic acid (SNPs: S4_16315718, S3_3964985, S4_16317194, S1_30882559, S7_39208926, S5_38026026) for harvest time point 3 under DS (Figure S6). In addition, significant MTAs with gene annotations that corresponded with and supported well the rest of our results were found for metabolites such as cyclic sugar alcohol (pinitol) (SNP: S4_27328584, harvest time point 1 under drought) and malic acid (SNPs: S2_32066110, S1_33885468 and S1_9042532, harvest time point 3 under drought). The gene annotations of significant MTAs identified in this study can be found in Table S7 and their relevance to chickpea drought tolerance is discussed below.

## Discussion

In the present study, 36 chickpea genotypes from different geographical locations were subjected to drought stress (DS) to evaluate their physiological and metabolomic responses. The study aims to uncover metabolite responses across three pod‐filling stages (i.e. three harvest time points) (Figure 1a) that contribute to drought stress tolerance and its impact on seed yield (Figure 2a). This approach has recently attracted more attention (Khan *et al*., 2019; Nisa *et al*., 2020; Singh *et al*., 2023). Due to our experimental setup, we also tested the adaptability of chickpea in a cooler European central climate compared to their place of origin. Identifying metabolite biomarkers that characterize the main drought response and differential metabolite fluxes across pod‐filling stages will enable the development of improved chickpea cultivars with increased drought tolerance and higher seed yield, which can be further incorporated into breeding programmes.

### Pod‐filling stages and photosynthetic activity as determinants for final yield performance under drought stress

Pods in legumes are fruit structures where the seeds develop (Gupta *et al*., 2016). The quantity and quality of nutrients transferred during the seed‐filling period greatly affect final seed weight and yield. Under water deficit conditions, crop growth and final grain yield at any developmental stage can be affected, particularly damaging the pod‐filling stage (Pushpavalli *et al*., 2015). Studies have shown that cultivars have differing pod‐filling potential (Gupta *et al*., 2016), suggesting the involvement of strong genetic components in this process. Therefore, in the present study, utilizing a range of 36 different chickpea genotype backgrounds and mapping their metabolomic changes during pod‐filling under DS can greatly advance our understanding of how genetic background influences final yield. For metabolomics analysis, primary leaves (close to pods) that are the source from where nutrients (such as sucrose, amino acids and fatty acids) are transported to the sink (endosperm) during seed developmental stages (Sehgal *et al*., 2018) were taken into consideration.

After the withdrawal of irrigation in the stress bed, soil water content (%) (Figure 1b) decreased linearly under the DS compared to WW condition and plateaued at day 19, after which it remained the same, maintaining equal levels of water deficiency across the three pod‐filling stages. The maximum photochemical efficiency of PSII (*F*_*v*_/*F*_*m*_) started decreasing rapidly after day 19. It reached a low level (0.4 *F*_*v*_/*F*_*m*_) by day 45 DASt (Figure 1d), indicating increasing plant stress between advancing pod‐filling stages. Relative chlorophyll content slightly differed between DS and WW conditions at day 19 (early pod‐filling stage), whereas it did not differ significantly during the mid and late pod‐filling stages (Figure 1d). Though not statistically significant, the clear trend of less chlorophyll content in the DS conditions might be used as a physiological bio‐indication for drought stress tolerance in chickpea and signifies the role of NUE in the stress response. However, this must be investigated in more detail in future studies.

In contrast, leaf temperature differential significantly increased after 24 DASt, with a large difference between DS and WW conditions at 45 DASt. Thus at a later stage of stress the relative higher cooling of WW is not a contrast, but goes along with the lack of soil water content under DS. Even though two physiological measurements (*F*_*v*_/*F*_*m*_ and leaf temperature differential) demonstrated increasing stress levels across pod‐filling stages (Figure 1d), the metabolome data indicated the mid pod‐filling stage (harvest time point 2) as the most drought‐responsive time point. This was evident from the PCA, where PC 1 showed the strongest separation between DS and WW conditions (Figure 4b) compared to harvest time point 1 and 3 (Figure S1). Hence, we categorized harvest time point 1 to be an early preparatory stage during pod‐filling, which metabolically primes the plant for the main transfer of nutrients from the primary leaves to the seed embryo, whereas, the second harvest time point signifies the plant's leaf metabolism to be most flexible and adaptive to DS. Finally, at the late pod‐filling stage (45 DASt; harvest time point 3), the primary leaves have adapted to DS and reset to a metabolic state closer to the WW condition. It also signifies the end of the transfer of nutrients to the seed embryo, in which the primary leaves no longer need to adapt to DS flexibly.

### L‐threonic acid, fructose, and sugar alcohols are potential metabolite markers for the main drought response in chickpea in their natural habitat

Focusing on harvest time point 2 as the pod‐filling stage where most drought responses occur in the metabolome between WW and DS, the metabolites with the highest loading scores (absolute value >0.1) at PC1 (23.12% variance) include organic acids such as L‐threonic acid, ribonic acid, citramalic acid, glyceric acid and unknown 2 (oxalic acid, level 2 identification), fructose, sugar alcohols and amines (unknown sugar alcohol 3, unknown sugar alcohol 4, unknown sugar amine, unknown sugar), as well as amino acids (serine, tyrosine, lysine and unknown amino acid 3 (ethanolamine, level 2 identification)) (Figure 4b). L‐threonic acid was found to have the largest fold change between DS and WW conditions, followed by unknown sugar alcohol 3 and unknown sugar and fructose (Figure 4c). Threonic acid, among other organic acids, was previously shown to accumulate in the kabuli variety under DS (Nisa *et al*., 2020). This organic acid accumulates after ascorbic acid degradation (Truffault *et al*., 2017), which is one of the strongest antioxidants that scavenge reactive oxygen species (ROS) overproduced under various stress conditions. The production of threonate from ascorbic acid strongly indicates the upregulation of the ascorbate pathway in chickpea under DS. Threarate, a product of threonic acid oxidation (Parsons *et al*., 2011), contributes to osmoregulation under DS (Guerrier *et al*., 2000). Conversely, the high relative abundance of fructose under DS indicates the accumulation of soluble sugars by starch breakdown to increase osmotic potential and cause more absorption of the scarce moisture in the soil (Camisón *et al*., 2020). Accumulation of sugars and a reset in the source‐sink carbon relation are always activated under DS. This prevents oxidation and dehydration of cell membranes, supports osmotic adjustment, and lowers plant photosynthetic rates. Fructose also links to the citric acid cycle (TCA) by producing 3‐phosphoglycerate converted to pyruvic acid via phosphoenolpyruvate. The latter connects to the shikimic acid pathway and the further production of aromatic compounds such as tyrosine that lead to flavonoid and secondary metabolite production.

Sugar alcohols (unknown sugar alcohols 1, 3 and 4, unknown sugar amine, unknown sugar and myo‐inositol, cyclic sugar alcohol (ononitol, level 2 identification), threitol and pinitol) were identified with higher relative abundance under DS compared to WW conditions in harvest time point 2 (Figure 4a). These photosynthetic by‐products are known for their osmoprotective effects under DS (Ahn *et al*., 2018; Singh *et al*., 2015), accounting for 50% of the total osmotic adjustment in chickpea (Amede and Schubert, 2003). Sugar alcohols can also contribute to yield tolerance under DS by providing alternative carbon sources after being transported to seed coats and accumulating their respective α‐galactosides in mature seeds (Obendorf and Górecki, 2012). This is in congruence with our finding of sugar alcohols being the most significant negatively correlated metabolites to the SSI values.

Most sugars and sugar alcohols demonstrated relatively low abundance in the early pod‐filling stage (Figure 4a, harvest time point 1) with a slight increase under DS (e.g. unknown sugar alcohol 3 and unknown sugar amine). The depletion of sugars in primary leaves at early pod‐filling stages signifies that the sink strength has been reduced due to increased sugar transport to the seed coats. Nevertheless, there is an increase of sugars under DS in harvest time point 3 (fructose, galactose, glucose, sucrose, myo‐inositol, cyclic sugar alcohol (ononitol, level 2 identification), pinitol, unknown sugar alcohol 4), reaching their respective control levels (Figure 4a). These results indicate that the primary leaves recovered from lower water potential during the earlier pod‐filling stages and restored metabolic processes to normal at the late pod‐filling stage. This recovery was also observed in bean tissues when exposed to DS (González‐Hernández *et al*., 2012) and corresponds well with the smaller separation between DS and WW conditions at harvest time point 3 compared to harvest time point 2 along PC1 (Figure S1), which is also a sign of metabolic recovery to control levels. The fast metabolic recovery by the late pod‐filling stage shows high adaptive potential mediated mainly within harvest time point 2. Hence, it is probably important for determining final yield productivity in chickpea.

Interestingly, a higher relative abundance of various amino acids (alanine, aspartic acid, glycine, methionine, phenylalanine, serine, threonine, valine and unknown amino acids 1 and 2) as well as organic acids (fumaric acid, lactic acid, aconitic acid, glycolic acid and unknown carbonic acids 1 and 3) was observed at the early pod‐filling stage (Figure 4a). Similar observations were derived from the recent findings by Zhang and co‐workers, where an increased concentration of amino acids and organic acids was identified in the early grain‐filling stages in wheat (Zhang *et al*., 2021). Similarly, in soybean late pod‐filling stage amino acid content declined due to their incorporation into storage proteins (Kambhampati *et al*., 2021).

### The differential Jacobian gives clues to differential metabolite fluxes between chickpea genotypes

For a more mechanistic understanding of how the metabolome contributes to differences in yield performance, comparisons between the Jacobian matrices of high‐performing (Q1) versus low‐performing (Q3) chickpea genotypes were uncovered by differential fluxes that contribute to drought tolerance mechanisms in chickpea (Figure 2b; Table S1). Focusing at harvest time point 2, the higher flux from fumarate to malate reaction could explain higher performance in Q1 genotypes as the accumulation of fumaric and malic acid has been associated with stomatal closure (Araujo *et al*., 2011) and maintenance of growth under severe DS (Ashrafi *et al*., 2018). The higher flux from cis‐aconitate to succinate in Q1 genotypes might signify higher activity of the aconitase enzyme that hydrates cis‐aconitate to the intermediate isocitrate. Aconitase is highly sensitive to oxidative stress (Lehmann *et al*., 2009) and controls many aspects of carbon metabolism (Carrari *et al*., 2003). The slightly higher flux of ononitol to myo‐inositol in Q1 genotypes points to the raffinose pathway, where ononitol forms methylated galactinol by reacting with UDP‐galactose, which then forms raffinose and myo‐inositol (Dong *et al*., 2013). The interconversion of maltose and glucose is differentially regulated between Q1 and Q3 genotypes. In Q3 genotypes, an inhibition of flux from maltose to glucose was observed, indicating that after the breakdown of starch to maltose, small sugar accumulation and transport, known to confer higher drought tolerance (Du *et al*., 2020; Maleckova and Ponnu, 2022), is occurring at lower rates (Figure 5).

Conversely, glucose to maltose interconversion in Q1 genotypes is inhibited, allowing flux to flow towards starch breakdown products and transport. Higher flux through the ethanolamine and serine reaction in Q1 genotypes points to drought tolerance mechanisms via glycine betaine, which requires serine and ethanolamine to be synthesized and is shown to be involved in ROS scavenging under various abiotic stresses (Dos Reis *et al*., 2012). Phenolic compounds tyrosine and phenylalanine (high loading scores for PC1 and PC2 at harvest time point 2 under DS) (Figure 4b) are known to contribute to drought tolerance mechanisms after upregulation of phenylpropanoid genes due to drought (Sharma *et al*., 2019). Flux towards these compounds is inhibited in Q3 genotypes at harvest time point 2. This agrees with higher shikimic acid concentrations in some Q1 genotypes at harvest time point 2 since shikimic acid is a precursor of these phenolic compounds. Flux towards methionine and ornithine, involved in the urea cycle, is also inhibited in Q3 genotypes. The urea cycle contributes to nitrogen remobilization, polyamine and proline production, contributing to abiotic stress tolerance (Zhang and Becker, 2015) and proper seed setting (Liu *et al*., 2018). Furthermore, we have determined differential fluxes of the metabolites between Q1 and Q3 chickpea genotypes in harvest time points 1 and 3, for more details, see Document S1 and Figure 5.

### mGWAS identifies genetic control of differential metabolite fluxes

Metabolomic genome‐wide association studies analysis uncovered genetic associations between SNPs and metabolites that gave clues to differential genetic control under DS and WW conditions across the three harvest time points. Genetic control identified through mGWAS complements metabolic control identified by the differential Jacobian analysis. In harvest time point 1, the highly activated flux between galactose and glucose (Figure 5) points to higher activity towards glycolysis under DS. SNP S4_27328584 on chromosome 4, which was found to be associated with pinitol and ascorbic acid in harvest time point 1 under DS (Document S1), is likely found within the gene coding for gibberellin 20 oxidase 2 (GA20ox2) or glyceraldehyde‐3‐phosphate dehydrogenase of plastid 1 (GAPDH) gene. SNP S5_10723119 on chromosome 5 (associated to galactaric acid, Figure S6) was found to reside in the gene encoding 1‐deoxy‐D‐xylulose 5‐phosphate synthase 1 (DXS1). Together, the highly associated SNPs at harvest time point 1 provide further evidence for upregulation of the glycolysis pathway leading to the plastidic 2C‐methyl‐d‐erythritol 4‐phosphate (MEP) pathway that results in gibberellin production (Estévez *et al*., 2001). GAPDH redirects flux towards plastidial glycolysis, where glyceraldehyde‐3‐phosphate (GAP) is normally converted to pyruvate. The DXS1 gene converts GAP to 1‐Deoxy‐D‐xylulose 5‐phosphate, which then enters the MEP pathway (Tian *et al*., 2022) and results in the production of gibberellins and ABA (Estévez *et al*., 2001). GA20ox was shown to regulate gibberellin concentration in many plant species and stimulated germination and flowering in *A*. *thaliana* (Xu *et al*., 1997). SNP S1_13338812 is significantly associated with unknown sugar amine (Figure S6) and annotated to the sucrose‐phosphatase 1 gene that participates in sucrose and starch metabolism. This could explain the increasing reaction rate from sucrose in Q1 genotypes within time point 1 (Figure 5).

At harvest time point 2, SNP S3_37239676 associated with asparagine (Figure S6) was found on chromosome 3 within the alpha‐carbonic anhydrase (CA) 2 gene that assimilates CO_2_ to HCO_3_– and produces malate. CA is known to regulate stomatal closure under various stresses (Polishchuk, 2021), and it connects well with the elevated fumarate to malate flux in Q1 genotypes at harvest time point 2 (Figure 5), that also controls stomatal conductance under stress. At harvest time point 3, SNP S1_33885468 on chromosome 1, associated with malic acid under drought, is found on a gene encoding sucrose synthase, which converts glucose to fructose and sucrose and agrees with the higher flux of the fructose‐sucrose reaction in Q1 genotypes (Figure 5). SNPs S4_16315718 and S3_3964985 on chromosome 4, associated with succinic acid (Figure S6), are found in the coding region of the myo‐inositol monophosphatase‐like 1 gene, which is involved in the recycling of D‐inositol products (Nourbakhsh *et al*., 2015). It can explain the dominant flux from ononitol to myo‐inositol in harvest time point 3 (Figure 5). SNP S2_32066110, associated with malic acid at harvest 3, is found within the coding region of an Early Response to Dehydration gene (Igamberdiev and Kleczkowski, 2018). ERD genes are known to be induced by drought stress in response to ABA signalling and confer drought tolerance (Wu *et al*., 2023). SNP S1_9042532, associated with malic acid at harvest time point 3, is within the coding region of a glutamate decarboxylase (GAD) gene, which catalyses the decarboxylation of glutamate to GABA (Rashmi *et al*., 2018). This agrees with the implied activity of GABA inferred from the differential Jacobian at harvest time point 3 and its role in grain‐filling (Document S1). SNP S5_38026026, associated with succinic acid within harvest time point 3 under drought, is annotated to a nitrate transporter gene known to confer higher crop yields through nitrogen use efficiency (Fan *et al*., 2017). Alternatively, this SNP is also annotated to a senescence‐associated gene. During the late pod‐filling stages, there is a decrease in nitrogen uptake, which is then remobilized from the leaves to the seeds. This process leads to leaf senescence (Hajibarat and Saidi, 2022) and an upregulation of genes involved in senescence‐related processes.

## Conclusion

**Figure 6:**
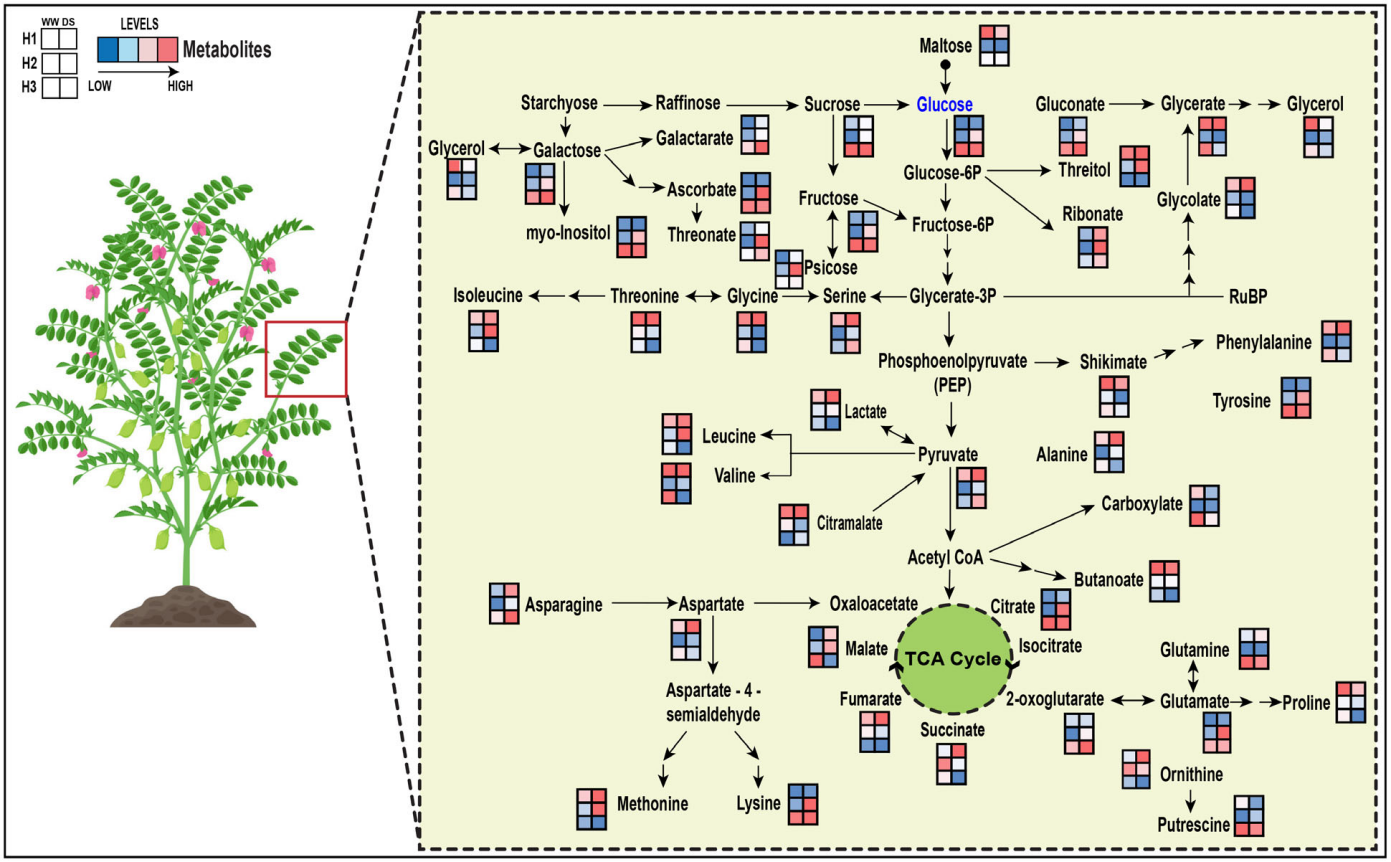
The biochemical pathway represents metabolic changes in chickpea leaves along three pod filling stage (i.e. 3 harvesting time points) under well‐watered (WW) and drought‐stress (DS) conditions. Metabolic changes are presented as mean relative abundance of all chickpea genotypes under each conditions. Values of metabolite levels from minimal to maximal are coloured from blue to red.

Large genotypic variation was observed among the chickpea germplasm subjected to drought stress, which underlines the usability of this collection for applied breeding programs. Using high‐throughput, non‐targeted GC–MS analysis, we identified and quantified 63 metabolites and their corresponding biochemical reactions within the chickpea leaf tissue under both DS and WW conditions in three distinct harvest time points (Figure 6). We identified L‐threonic acid, fructose and various sugar alcohols to be involved in the main adaptive drought response of chickpea at the mid‐pod‐filling stage (i.e. harvest time point 2). Different relative abundances of metabolites between high‐ and low‐performing genotypes at harvest time point 2 also include citric acid, glutamic acid, ribonic acid, shikimic acid, serine and glycerate as potential biomarkers for high seed yield, which points towards the activation of the central metabolism such as TCA cycle, GABA biosynthesis, and flavonoid pathways. The differential Jacobian analysis further established increasing fluxes towards glycolysis in harvest time point 1 and differential regulation of sugar alcohols, possibly depending on the level of sucrose concentration, leading to stachyose synthesis in Q1 genotypes. Inositol and sugar alcohol interconversions were also highly active at harvest point 2.

In contrast, the implied role of GABA and nitrogen remobilization is evident in differential fluxes in harvest time points 2 and 3, physiological measurements (relative chlorophyll content), and metabolite abundances, further supporting our mGWAS analysis. Taken together, the role of nitrogen metabolism in maintaining high seed yield performance in nitrogen‐fixing legumes such as chickpea is crucial. Accordingly, in follow‐up studies, we will address in more detail the relation of drought stress to pod filling and protein content (Benali *et al*., 2023; Cohen *et al*., 2021). Our differential Jacobian analysis further unveiled the interplay between various metabolic pathways across three‐time points in maintaining a good drought response and yield, which would not be possible by only looking at changes in concentrations of the metabolites alone. We would thus like to encourage and support further use of more advanced data‐driven mathematical tools, like the Jacobian matrix, in discerning and facilitating an all‐round understanding of adaptive, dynamic changes in metabolic and other molecular networks. Importantly, this study unveiled dynamic metabolite changes across the pod‐filling trajectory of chickpea, with important metabolite biomarkers that contribute to drought tolerance mechanisms and which can be translated to other legume crops. It also sheds light on the specific developmental stages where these biomarkers are most effective, which is important in their correct implementation to achieve a final high seed yield performance under drought. Furthermore, based on the SSI, promising genotypes are identified in this study that can serve as potential donors for designing future drought‐tolerant chickpea.

## Materials and methods

### Experimental design, growth conditions and drought treatment

Thirty‐six chickpea (*Cicer arietinum* L.) genotypes from different geographical origins were selected for this study. These genotypes were part of the global composite collection (Varshney *et al*., 2021c) obtained from the gene bank repository of the International Crops Research Institute for the Semi‐Arid Tropics (ICRISAT), India. The field study was conducted at the Augarten experimental garden at the University of Vienna, Vienna, Austria (48°13′26.2″ N 16°22′29.1″ E). The experiment was started in June and concluded in October. Seeds were hand‐sown in the experimental plot. The distance between rows was 30 cm with 20 cm between plants, and the sowing depth was 3–5 cm with four to five seeds sown per genotype per replicate. The experiment was laid out in a randomized manner with three biological replicates per genotype. The irrigation was adapted to the plant's physiological needs. The difference in the soil water content between control (WW, well‐watered) and stressed plants (DS, drought stress) was the first indication of drought imposed; at this stage, plants were at 50% flowering stage. Photosynthetic activities were estimated until 45 days after the stress (Figure 1a). Leaf tissues were harvested for metabolomics at three‐time points (representing three pod‐filling stages): 19, 24 and 45 days after stress (DASt) (Figure 1a). The harvested samples were frozen in liquid nitrogen to stop any enzymatic activity. The tissue samples were grounded in liquid nitrogen using mortar and pestle. Pulverized tissue was stored at −80 °C until further analysis. The plants were allowed to grow till the senescence stage (120 days) under control and stress conditions to evaluate the impact of drought stress on yield.

### *In vivo* measurements of photosynthetic activity and microclimate conditions

Soil water content was monitored using Delta T theta probe ML2 in close vicinity of the plant roots (between 10 and 40 cm of soil depth) of the soil in the experimental plot (Table S1; Figure 1b). Plant height was measured using a folding yardstick to determine the growth and development of the plants under WW and DS conditions (Table S1; Figure 1c).

The photosynthetic activity was measured using PhotosynQ V2.0 (https://www.photosynq.com/technology), a cloud‐based, integrated system using microcontrollers (Arduino‐based) in a non‐destructive manner (Table S1; Figure 1d). Parameters such as relative chlorophyll content (at an absorbance at 430 nm and 560 nm), chlorophyll fluorescence (*F*_*v*_/*F*_*m*_ ratio), and leaf temperature differential (related to ambient temperature, evapotranspiration cooling of the plant leaf by stomatal conductance and soil evapotranspiration respectively) were determined for each genotype under WW and DS condition (Table S1; Figure 1d).

### Stress susceptibility index

The SSI was determined to evaluate drought tolerance and to differentiate chickpea genotypes on the degree of drought tolerance. SSI was calculated using 100‐seed weight (Table S1), allowing access to the seed quality and grain yield potential under drought conditions. For each genotype, the SSI was calculated, according to Fischer and Maurer, as differences in the results obtained for drought stress (DS samples) and control (WW samples) conditions by using the following equation: SSI = [1 − Yp/Ys]/SI; SI = [1 − MYs/MYp], where Yp is the mean value for the investigated trait under WW conditions, Ys is the mean trait value under DS condition, MYp is the mean trait value of all investigated genotypes under WW conditions respectively, MYs is the mean trait value of all genotypes under DS conditions, respectively and SI represents stress intensity (Fischer, 1998) (Table S1).

### Metabolomics using gas chromatography coupled to mass spectrometry (GC–MS)

Metabolomic analysis was performed according to Weckwerth *et al*. (2004). The leaf tissues were freeze‐dried in liquid nitrogen (N_2_) and homogenized using mortar and pestle. Metabolites were extracted with 500 μL pre‐cooled extraction solution of methanol: chloroform: water (2.5:2:1 v/v/v). The extracts were vortexed, incubated for 8 min on ice and then centrifuged for 4 min at 20 000 ***g*** and 4 °C. The supernatant was removed and transferred to an Eppendorf tube (2 mL). The metabolites were once again extracted by adding another 500 μL pre‐cooled extraction solution (methanol:chloroform:water (2.5:2:1 v/v/v)) on the pellet. Further, distilled water (300 μL) was added, and the solution was then centrifuged for 2 min at 20 000 ***g***/4 °C to obtain the phase separation. The upper polar phase (methanol/water) was combined with the first extraction supernatant, then dried in the speed vac (SCANVAC Cool Safe 110‐4, SpeedVacuum concentrator; Labogene) and stored at −80 °C until further derivatization. For derivatization, the dried polar phase was dissolved in 20 μL methoxylamine hydrochloride in pyridine (40 mg/mL) and incubated for 90 min at 30 °C in a thermoshaker. Eighty microliters of N‐methyl‐N‐(trimethylsilyl) trifluoroacetamide (MSTFA) (Macherey Nagel, Germany) was added in all the samples, which were incubated at 37 °C for 30 min in a thermoshaker. After the incubation, the samples were centrifuged for 2 min at 14 000 ***g***, transferred to GC‐micro vials with micro inserts, and closed with crimp caps. Along with the samples, a 60 μL retention index marker solution of even alkanes from C10 to C40 in hexane (Sigma‐Aldrich) at a concentration of 50 mg/L was also prepared with MSTFA spiked.

GC–MS analyses of primary metabolites, a LECO Pegasus 4D GC × GC TOF‐MS instrument was used. Samples, alkanes, and blanks were injected with a split/splitless injector at the constant temperature of 230 °C. The injection volume was 1 μL of the derivatized sample; the injection was performed at a split ratio of 1:5 and 1:100. GC separation was conducted on an HP‐5MS column (30 m × 0.25 mm × 0.25 mm; Agilent Technologies) using helium as carrier gas at a flow rate of 1 mL/min. The temperature gradient started at 70 °C isothermal for 1 min, followed by a heating ramp of 9 °C/min to 330 °C, where the temperature was held for 7 min. The transfer line temperature was 250 °C, and the ion source temperature was set to 200 °C. Mass spectra were acquired with an acquisition rate of 20 spectra/s at an m/z range of 40–600 using a detector voltage of 1500 V and an electron impact ionization of 70 eV.

Data analysis was performed with Chroma TOF software (Leco, Mönchengladbach, Germany). Briefly, representative chromatograms of different samples were used to generate a reference peak list based on quality control samples (QC) including reference compounds, and all other data files were processed against this reference list. Deconvoluted mass spectra were matched against an in‐house mass spectral library. Peak annotations and peak integrations were checked manually before exporting peak areas for relative quantification. The internal standard and the fresh weight of the sample were used to normalize the peak areas. Metabolite amounts are given in arbitrary units corresponding to the peak areas of the chromatograms (Ghatak *et al*., 2022; Zhang *et al*., 2021, 2024).

### Bioinformatics and statistics analysis

Multivariate (principal component analysis (PCA)) analysis was performed using the R program (v 4.0.2) (pRocessomics, https://github.com/Valledor/pRocessomics). Heatmaps, partial least squares‐discriminant analysis (PLS‐DA) and k‐means cluster analysis were computed and constructed using the R package (pRocessomics). Seed yield and metabolite profile were correlated by simple Pearson correlation using the SSI values and log‐transformed relative abundance values of all metabolites identified under DS at the second harvest time point. The R function ‘cor.test’ (stats basic package in R, R version 3.6.1) was used to calculate the Pearson correlation between a single metabolite and SSI for each chickpea genotype. The one‐way analysis of variance (ANOVA) was applied to compare the agronomy and physiology data, including soil water content, plant height, *F*_*v*_/*F*_*m*_, relative chlorophyll content and leaf temperature difference, between WW and DS conditions, using the ‘aov’ function from the stats basic package in R (R version 3.6.1).

### Data‐driven inverse mathematical modelling approach

For the calculation of the Jacobian matrix, an inverse data‐driven method was used that only requires the covariance matrix from the data and an arbitrary noise matrix as inputs. This was previously developed by (Sun and Weckwerth, 2012) using the Lyapunov equation (Weckwerth, 2019):

(2) $$\mathrm{JC}+{\mathrm{CJ}}^{T}=-2D,$$

where *C* is the covariance matrix, *D* is the noise matrix, *T* stands for transpose of a matrix and *J* is the vectorized form of the Jacobian matrix. Input data for the covariance matrix included 48 metabolites (all known metabolites as well as unknowns with level 2 identification using the NIST library) and was log‐transformed separately for the Q1 and Q3 genotypes and at each time point. The noise matrix D was arbitrarily set with diagonal entries randomly drawn from a standard normal distribution and non‐diagonal entries set to 0. A reconstructed metabolic network of central metabolism in chickpea was used as a constraint using SIM‐network settings with cost 3 (Li *et al*., 2023) as well as manual curation with the KEGG pathway database as a reference. The calculation was repeated 10^4^ times and the median value was taken for the final output.

The Jacobian matrices between Q1 and Q3 genotypes were compared using the differential Jacobian. This was previously established (Nagele *et al*., 2014), as in:

(3) $$d\left(\mathrm{Jij}\right)=\log\left|\frac{\frac{\partial f\;A,C2}{\partial \;A,C2}}{\frac{\partial f\;A,C1}{\partial \;A,C1}}\right|$$

where the numerator is a Jacobian entry from one condition (Q1 genotypes) and the denominator is the same Jacobian entry from the other condition (Q3 genotypes). Positive differential entries indicate larger fluxes in one condition whereas negative differential entries indicate higher fluxes for the other condition and give clues to underlying regulatory differences at the level of proteins or transcripts. All calculations were performed in MATLAB. Circular plots of differential Jacobian entries for each time point were also plotted using MATLAB. MATLAB scripts are either downloadable as a GUI toolbox COVAIN (https://mosys.univie.ac.at/resources/software/) and additional scripts from Li and co‐workers are available by request (Li *et al*., 2023).

### Metabolic GWAS and genomic prediction

The raw genotypic data for the 36 genotypes were obtained from the chickpea 3366 Genomes Project (Varshney *et al*., 2021c). The genotypic data were filtered based on MAF cut‐off ≥0.05, a missing rate of ≤20% and a heterozygosity rate of ≤20%, using vcftools v0.1.16 (Danecek *et al*., 2011) to obtain a set of 153 820 high‐quality SNPs. The metabolomic data were averaged across the three biological replicates. The averaged metabolomic data (after log‐transformation) were checked for normal distribution using shapiro.test function in R. If the metabolite did not follow normal distribution (*P*‐value cut‐off 0.05) for a given harvest time point, the GWAS analysis was not performed for the respective metabolite. The filtered genotypic data and metabolite data were subjected to genome‐wide association studies (GWAS) analysis with GAPIT R package using FarmCPU method (Liu *et al*., 2016) as described in (Garg *et al*., 2022). The GWAS analysis was performed separately for all metabolites under each treatment (WW and DS) and harvest time point. The significant marker‐trait associations were obtained based on a *P*‐value cut‐off of 1E‐05. Additionally, for gene annotation, the FASTA DNA sequences 5000 bp upstream and downstream of the identified significant SNPs were retrieved and matched against homologous *A*. *thaliana* annotated gene sequences using the blast search tool of EnsemblPlants. Overlapping genes with the retrieved sequences were chosen based on overall score according to relatively low *E*‐values and high % identity similarity.

## Funding

PC and IP are thankful to the Austrian Science Fund (FWF), Grant agreement number I 5234 for their support. AG is thankful to the Vienna Metabolomics Center (VIME), Grantham foundations and European Union Horizon 2020 research and innovation program under grant agreement number GA 2020 862‐858 (ADAPT). SZ is supported by FFG – The Austrian Research Promotion Agency, Vienna, Austria.

## Conflict of interest

The authors declare no competing financial interest.

## Author contributions

Conceived and designed the experiments: PC, AG and WW; Performed the field experiment: PC, AG, AB, GB and WW; Performed GC–MS measurements: PC, IP and AG; Analysed GC–MS raw data: IP and PC; Analysed the data: PC, CLH, IP, AG, VG, SZ, RB, LV, RKV and WW; Drafted the manuscript: PC, IP and AG; Edited the manuscript: PC and WW. All the authors read and agreed on the final version of the manuscript.

## Supporting information


**Data S1** Differential Jacobian results and discussion for time points 1 and 3.

**Figure S1** PCA Score plot and PC1 and PC2 top‐ranked metabolites. (a) Harvest 1 and (b) Harvest 3. (a, b) Top 20 scoring loadings (10 highest and 10 lowest) of PC1 and PC2 are shown by row for each PCA, bar colours indicate the experimental condition in which each top‐scoring metabolite is more accumulated. Ellipses showing different colours indicate different experimental conditions (*n* = 108 biologically independent replicates).

**Figure S2** PLS‐DA Score plot and PC1 and PC2 top‐ranked metabolites. (a) Harvest 1, (b) Harvest 2 and (c) Harvest 3. (a, b) Top 20 scoring loadings (10 highest and 10 lowest) of component 1 and component 2 are shown by row for each PLS‐DA, bar colours indicate the experimental condition in which each top‐scoring metabolite is more accumulated. Ellipses show a 90% confidence interval. Different colours indicate different experimental conditions (*n* = 108 biologically independent replicates).

**Figure S3** Primary metabolites identified in chickpea. Bar plots representing the relative abundance of primary metabolites measured in chickpea leaves at harvest time point 2. The bar plots show the average, standard error and each independent value in circles. The colour of the bar plots represents the experimental condition (WW, well‐watered and DS, drought‐stressed samples).

**Figure S4** (a) Harvest 1, and (b) Harvest 3. Hierarchically clustered heatmap of the 36‐chickpea genotypes using the top 20 metabolites with higher loadings in the first component of PLS‐DA. The bi‐clustering uses averages linkage of Pearson correlation distance between chickpea genotypes and metabolites. Metabolic changes are presented as means of three replicates. Colours indicate increases (red) and decreases (blue).

**Figure S5** K‐means clustering and chemical family pie chart. K‐means clustering of needle metabolites of each experimental condition in each harvest time. 63 detected metabolites in all the treatments were scaled in each dataset. Metabolites were grouped in 10 clusters based on the accumulation pattern occurring during experiment days (19, 24 and 45 DASt). Colours indicate treatment level: green (WW, well‐watered samples) and yellow (DS, drought‐stressed samples). The most intense solid line shows the mean for each cluster, and the light lines show individual patterns. Pie charts of each metabolic pathway of annotated metabolites for each cluster. Different colours indicate different chemical family (organic acids, amino acids, sugars, sugars alcohols, amines and unknowns).

**Figure S6** mGWAS analysis. Selected Manhattan plots and QQ plots show association with the metabolites under well‐watered (WW) and drought‐stress (DS) conditions in three harvesting time points (19, 24 and 45 DASt). The black horizontal line in the Manhattan plots represents the significance threshold of *P*‐value = 1e–5.

**Table S1** (A) Photosynthetic activity under WW and DS in chickpea genotypes. (B) Seed yield parameters under WW and DS in chickpea genotypes. (C) Soil water content (%) and plant height measurements under WW and DS in chickpea genotypes. (D) Stress Susceptibility Index (SSI) calculation for 36 chickpea genotypes under WW and DS conditions. G (Genotypes; 1–36), WW (well‐watered) and DS (drought‐stressed).

**Table S2** (A) The list identified metabolites in Harvest time point 1 and their relative abundance values for each chickpea genotype and experimental conditions. (B) The list identified metabolites in Harvest time point 2 and their relative abundance values for each chickpea genotype and experimental conditions. (C) The list identified metabolites in Harvest time point 3 and their relative abundance values for each chickpea genotype and experimental conditions. (D) Univariate analysis of metabolites. For each metabolite, mean and standard deviation (SD) are provided. ANOVA pairwise comparisons were performed for WW and DS samples at each harvest time point, and *P*‐values obtained after ANOVA were provided. (E) Chemical family classification table. Metabolic changes in chickpea leaves along three harvest time points under well‐watered and drought‐stressed conditions. Metabolic changes are presented as relative abundance means for each treatment. For each chemical family, mean values were summed. G (Genotypes; 1–36), WW (well‐watered) and DS (drought‐stressed).

**Table S3** PCA loadings of chickpea genotypes under WW and DS conditions for three harvesting time points.

**Table S4** PLS‐DA analysis of chickpea genotypes under WW and DS conditions for three harvesting time points.

**Table S5** Cluster analysis of chickpea genotypes using *K*_means_ analysis under WW and DS conditions for three harvesting time points.

**Table S6** List of metabolites included in the differential Jacobian analysis with their level of identifications.

**Table S7** mGWAS analysis. Details of genetic associations between SNPs and metabolites of chickpea genotypes under control and drought stress conditions for three harvesting time points, including gene annotations. H (harvest time point), C (control) and D (drought).

## Data Availability

The data that supports the findings of this study are available in the supplementary material of this article.
