# Supplementary material for: Natural variation in the chickpea metabolome under drought stress
Source: Plant Biotechnol J. 2024 Oct 16;22(12):3278–94. doi: 10.1111/pbi.14447 (PMC11606430; doi:10.1111/pbi.14447)
Supplement: Supplementary file 3 — Figure S2 PLS‐DA Score plot and PC1 and PC2 top‐ranked metabolites. (a) Harvest 1, (b) Harvest 2 and (c) Harvest 3. (a, b) Top 20 scoring loadings (10 highest and 10 lowest) of component 1 and component 2 are shown by row for each PLS‐DA, bar colours indicate the experimental condition in which each top‐scoring metabolite is more accumulated. Ellipses show a 90% confidence interval. Different colours indicate different experimental conditions (n = 108 biologically independent replicates). [file PBI-22-3278-s010.docx]

**a) Harvest 1**

**DA Maximum Distance Plot**

5

0

-5

**PLS-DA Component 1 top scoring variables** Shikimic acid

Proline

Unknown sugar C5

Butanoic acid

Carboxylic acid

Putrescine

Unknown amino acid 3

Maltose

Unknown carbonic acid 3

L-Threonic acid

Unknown sugar

Galactaric acid

Malic acid

Gluconic acid

Ribonic acid

Unknown sugar alcohol 3

Glycolic acid

Glycerol

Galactose

**PLS-DA Component 2 top scoring variables** Glycolic acid

Unknown carbonic acid 1

Citric acid

Glycine

Lactic acid

Unknown sugar alcohol 2

Ornithine

Galactose

Unknown sugar

Unknown 1

Putrescine

Phenylalanine

Unkown 2

Citramalic acid

Isoleucine

Proline

Valine

Threonine

Butanoic acid

-10

-10 -5 0 5

Component 1 - 16.62 % of variance

Unkown 2

−0.3 −0.2

−0.1 0 0.1 0.2 0.3

Loadings

Glycerol

−0.25 −0.2 −0.15

−0.1 −0.05 0 0.05 0.1

Loadings

**b) Harvest 2**

**DA Maximum Distance Plot** **PLS-DA Component 1 top scoring variables** **PLS-DA Component 2 top scoring variables**

5

0

-5

-10 -5 0 5

Component 1 - 21.64 % of variance

Threonine

Maltose

Unkown 2

Glycine

Unknown sugar alcohol 2

Glyceric acid

Shikimic acid

Serine

Unknown amino acid 3

Fructose

Citric acid

Unknown sugar amine

Glutamic acid

Unknown sugar alcohol 1

Citramalic acid

Unknown sugar alcohol 4

Ribonic acid

Unknown sugar

Unknown sugar alcohol 3

L-Threonic acid

−0.3

−0.2 −0.1 0 0.1 0.2

Loadings

Ribonic acid

Unknown sugar alcohol 3

Citric acid

Unknown sugar

L-Threonic acid

Malic acid

Unknown 1

Lactic acid

Unknown carbonic acid 2

Alanine

Unknown amino acid 3

Psicose

Glucose

Lysine

Tyrosine

Galactose

Citramalic acid

Cyclic sugar alcohol

Putrescine

Phenylalanine

−0.1

0 0.1

Loadings

0.2

**c) Harvest 3**

**DA Maximum Distance Plot** **PLS-DA Component 1 top scoring variables** **PLS-DA Component 2 top scoring variables**

10

5

0

-5

-5 0 5 Component 1 - 16.71 % of variance

**Treatments**

90 % conﬁdence interval; Harvest 2 - WW 90 % conﬁdence interval; Harvest 2 - DS

DS

Galactaric acid

Unknown amino acid 3

Unknown sugar amine

Unknown sugar alcohol 4

Unknown sugar

Ribonic acid

L-Threonic acid

Unknown sugar alcohol 3

Psicose

Pyruvic acid

Leucine

Isoleucine

Proline

Succinic acid

Valine

Glycine

Lactic acid

Threonine

Malic acid

Unkown 2

−0.1 0 0.1 0.2 0.3 Loadings

Unknown sugar alcohol 1

Galactaric acid

Maltose

Unkown 2

Lactic acid

Ascorbic acid

2-oxo-Glutaric acid

Malic acid

Cyclic sugar alcohol

Threitol

Unknown sugar alcohol 2

Phenylalanine

Ribonic acid

Asparagine

Glutamine

Unknown amino acid 1

Glycolic acid

Unknown amino acid 3

Unknown carbonic acid 2

Unknown sugar alcohol 3

−0.1 0 0.1 0.2 0.3 Loadings

**Figure S2.** PLS-DA Score plot and PC1 and PC2 top-ranked metabolites. (a) Harvest 1, (b) Harvest 2 and (c) Harvest 3 (a-c) Top 20 scoring loadings (10 highest and 10 lowest) of component 1 and com-ponent 2 are shown by row for each PLS-DA, bar colours indicate the experimental condition in which each top-scoring metabolite is more accumulated. Ellipses show a 90% confidence interval. Different colours indicate different experimental conditions (n = 108 biologically independent replicates).
