# Supplementary material for: Natural variation in the chickpea metabolome under drought stress
Source: Plant Biotechnol J. 2024 Oct 16;22(12):3278–94. doi: 10.1111/pbi.14447 (PMC11606430; doi:10.1111/pbi.14447)
Supplement: Supplementary file 5 — Figure S4 (a) Harvest 1, and (b) Harvest 3. Hierarchically clustered heatmap of the 36‐chickpea genotypes using the top 20 metabolites with higher loadings in the first component of PLS‐DA. The bi‐clustering uses averages linkage of Pearson correlation distance between chickpea genotypes and metabolites. Metabolic changes are presented as means of three replicates. Colours indicate increases (red) and decreases (blue). [file PBI-22-3278-s013.docx]

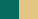

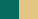


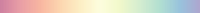

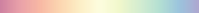


**Figure S4.** (a) Harvest 1, and (b) Harvest 3. Hierarchically clustered heatmap of the 36-chickpea genotypes using the top 20 metabolites with higher loadings in the first component of PLS-DA. The bi-clustering uses averages linkage of Pearson correlation distance between chickpea genotypes and metabolites. Metabolic changes are presented as means of three replicates. Colours indicate increases (red) and decreases (blue).

G31- Harvest 1- WW G29- Harvest 1- WW G25- Harvest 1- WW G25- Harvest 1- DS G27- Harvest 1- WW G26- Harvest 1- WW G33- Harvest 1- WW G36- Harvest 1- WW G35- Harvest 1- WW G23- Harvest 1- WW G30- Harvest 1- WW G21- Harvest 1- WW G32- Harvest 1- WW G24- Harvest 1- WW G22- Harvest 1- WW G28- Harvest 1- WW G20- Harvest 1- WW G19- Harvest 1- WW G15- Harvest 1- WW G9- Harvest 1- WW G11 - Harvest 1 - WW G1- Harvest 1- WW G7- Harvest 1- WW G5- Harvest 1- WW G10- Harvest 1- WW G6- Harvest 1- WW G17- Harvest 1- WW G3- Harvest 1- WW G18- Harvest 1- WW G16- Harvest 1- WW G8- Harvest 1- WW G14- Harvest 1- WW G12- Harvest 1- WW G13- Harvest 1- WW G4- Harvest 1- WW G36- Harvest 1- DS G35- Harvest 1- DS G29- Harvest 1- DS G17- Harvest 1- DS G16- Harvest 1- DS G19- Harvest 1- DS G31- Harvest 1- DS G28- Harvest 1- DS G22- Harvest 1- DS G24- Harvest 1- DS G21- Harvest 1- DS G32- Harvest 1- DS G23- Harvest 1- DS G30- Harvest 1- DS G34- Harvest 1- DS G33- Harvest 1- DS G2- Harvest 1- DS G2- Harvest 1- WW G1- Harvest 1- DS G34- Harvest 1- WW G15- Harvest 1- DS G4- Harvest 1- DS G8- Harvest 1- DS G5- Harvest 1- DS G3- Harvest 1- DS G20- Harvest 1- DS G11 - Harvest 1 - DS G10- Harvest 1- DS G13- Harvest 1- DS G7- Harvest 1- DS G6- Harvest 1- DS G27- Harvest 1- DS G26- Harvest 1- DS G18- Harvest 1- DS G14- Harvest 1- DS G12- Harvest 1- DS G9- Harvest 1- DS


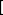

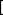

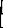

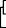

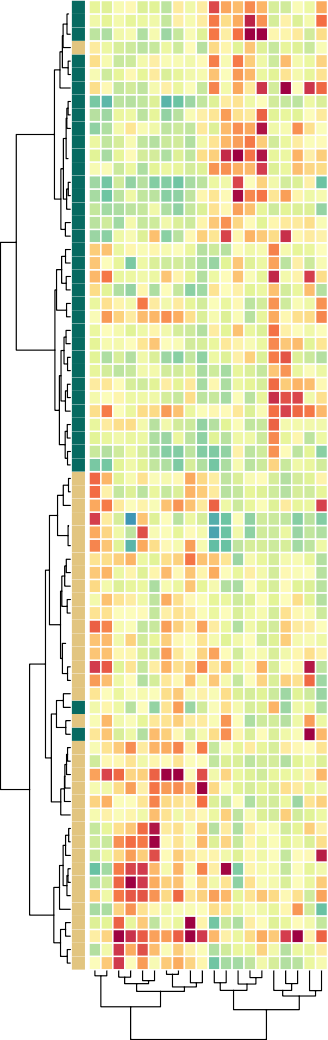

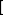

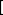

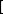

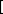

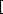

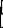

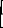

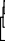

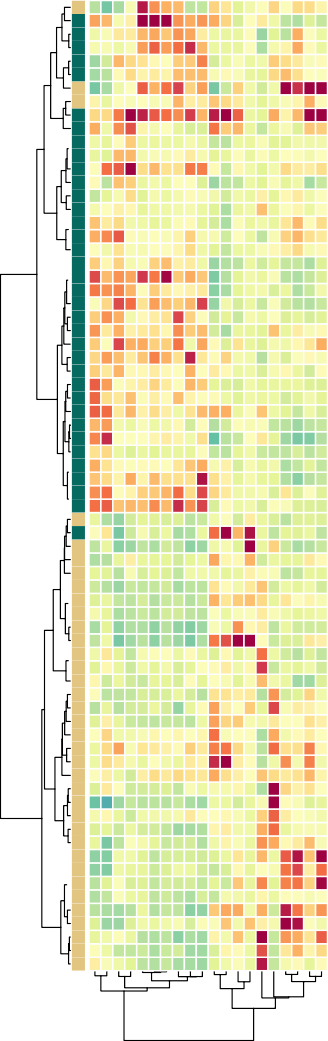


**a) Harvest 1**

3

**Treatment** WW DS

**Treatment**

Unknown sugar L-Threonic acid Gluconic acid Malic acid Galactaric acid

Unknown carbonic acid 3 Unknown sugar alcohol 3 Ribonic acid

Galactose Glycolic acid

Unknown sugar C5 Shikimic acid Unkown 2

Maltose

Unknown amino acid 3 Glycerol

Putrescine Proline Carboxylic acid Butanoic acid

2

1

0

-1

-2

-3

**b) Harvest 3**

3

**Treatment** WW DS

**Treatment**

Malic acid Succinic acid Unkown 2

Lactic acid Leucine Valine

Isoleucine Proline

Threonine Glycine Ribonic acid

Unknown sugar alcohol 4 L-Threonic acid Galactaric acid

Pyruvic acid

Unknown amino acid 3 Psicose

Unknown sugar amine Unknown sugar alcohol 3 Unknown sugar

2

1

0

-1

-2

-3

G13&Harvest3_DS G13&Harvest3_WW G24&Harvest3_WW G18&Harvest3_WW G34&Harvest3_WW G33&Harvest3_WW G18&Harvest3_DS G4&Harvest3_DS G14&Harvest3_WW G12&Harvest3_WW G36&Harvest3_WW G35&Harvest3_WW G2&Harvest3_WW G25&Harvest3_WW G32&Harvest3_WW G31&Harvest3_WW G29&Harvest3_WW G28&Harvest3_WW G30&Harvest3_WW G27&Harvest3_WW G4&Harvest3_WW G3&Harvest3_WW G1&Harvest3_WW G26&Harvest3_WW G17&Harvest3_WW G7&Harvest3_WW G23&Harvest3_WW G21&Harvest3_WW G22&Harvest3_WW G16&Harvest3_WW G15&Harvest3_WW G6&Harvest3_WW G5&Harvest3_WW G19&Harvest3_WW G20&Harvest3_WW G9&Harvest3_WW G11&Harvest3_WW G8&Harvest3_WW G12&Harvest3_DS G10&Harvest3_WW G27&Harvest3_DS G9&Harvest3_DS G17&Harvest3_DS G16&Harvest3_DS G3&Harvest3_DS G22&Harvest3_DS G19&Harvest3_DS G6&Harvest3_DS G20&Harvest3_DS G10&Harvest3_DS G21&Harvest3_DS G24&Harvest3_DS G7&Harvest3_DS G5&Harvest3_DS G2&Harvest3_DS G11&Harvest3_DS G8&Harvest3_DS G15&Harvest3_DS G28&Harvest3_DS G25&Harvest3_DS G23&Harvest3_DS G26&Harvest3_DS G14&Harvest3_DS G33&Harvest3_DS G32&Harvest3_DS G34&Harvest3_DS G30&Harvest3_DS G29&Harvest3_DS G1&Harvest3_DS G36&Harvest3_DS G31&Harvest3_DS G35- Harvest 3- DS
