# Supplementary material for: Natural variation in the chickpea metabolome under drought stress
Source: Plant Biotechnol J. 2024 Oct 16;22(12):3278–94. doi: 10.1111/pbi.14447 (PMC11606430; doi:10.1111/pbi.14447)
Supplement: Supplementary file 7 — Figure S6 mGWAS analysis. Selected Manhattan plots and QQ plots show association with the metabolites under well‐watered (WW) and drought‐stress (DS) conditions in three harvesting time points (19, 24 and 45 DASt). The black horizontal line in the Manhattan plots represents the significance threshold of P‐value = 1e–5. [file PBI-22-3278-s011.docx]

WW DS

Galactaric acid - harvest time point 1 Galactaric acid - harvest time point 1

Observed -log10(*p*)

Observed -log10(*p*)

Expected -log10(*p*)

-log10(*p*)

-log10(*p*)

Expected -log10(*p*)

Chromosome

Chromosome

Unknown sugar amine - harvest time point 1 Unknown sugar amine - harvest time point 1

Observed -log10(*p*)

Expected -log10(*p*)

-log10(*p*)

-log10(*p*)

Observed -log10(*p*)

Expected -log10(*p*)

Chromosome Chromosome

Asparagine - harvest time point 2 Asparagine - harvest time point 2

Observed -log10(*p*)

Expected -log10(*p*) Expected -log10(*p*)

-log10(*p*)

-log10(*p*)

Observed -log10(*p*)

Chromosome Chromosome

Unknown sugar alcohol 4 - harvest time point 2 Unknown sugar alcohol 4 - harvest time point 2

Observed -log

10(*p*)

Expected -log10(*p*)

-log10(*p*)

-log10(*p*)

Observed -log10(*p*)

Expected -log10(*p*)

Chromosome Chromosome

Succinic acid - harvest time point 3 Succinic acid - harvest time point 3

10(*p*)

Expected -log10(*p*)

-log10(*p*)

Observed -log

-log10(*p*)

Observed -log10(*p*)

Expected -log10(*p*)


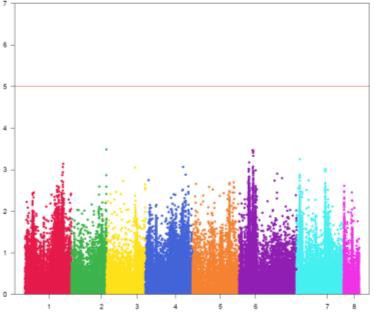

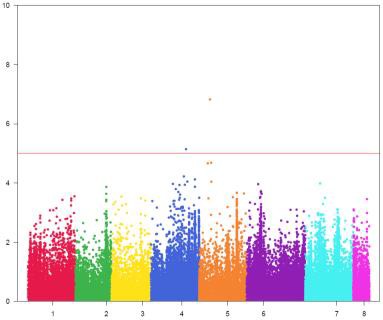

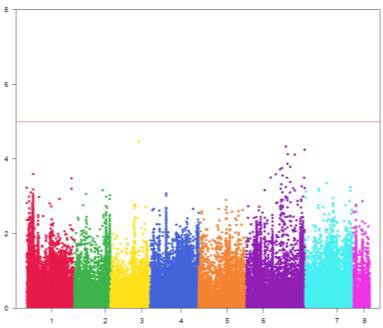

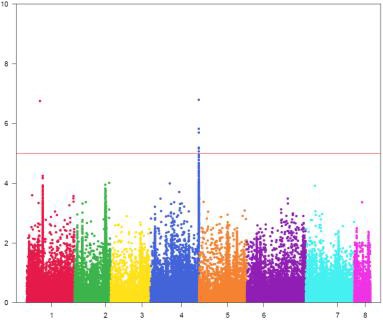

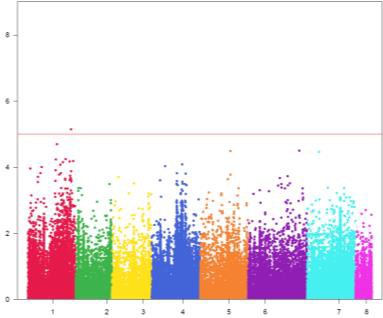

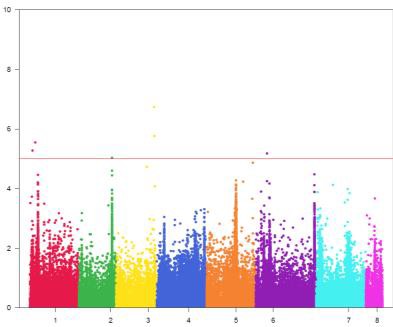

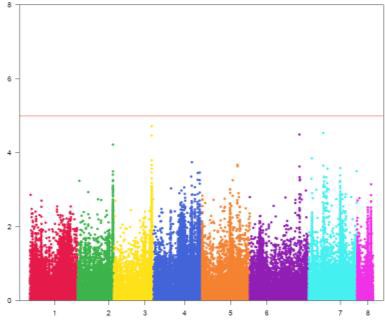

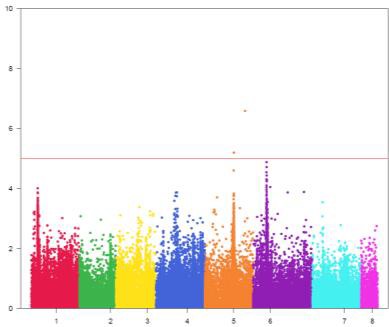

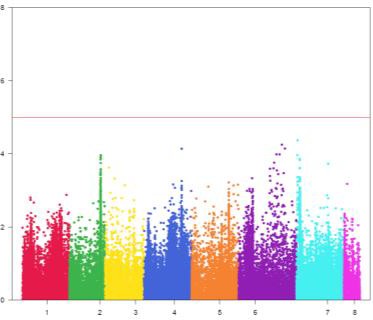

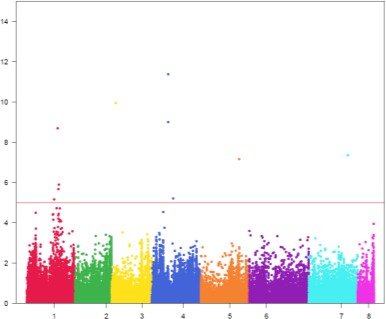

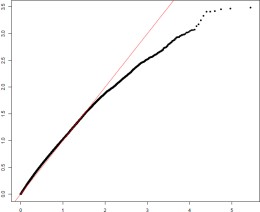

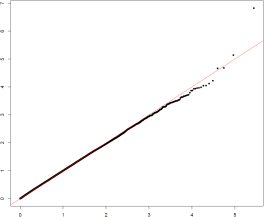

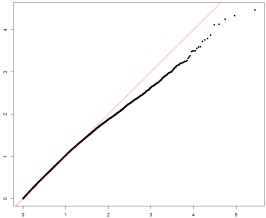

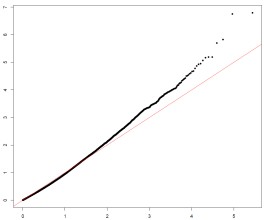

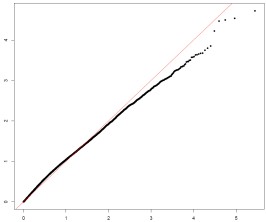

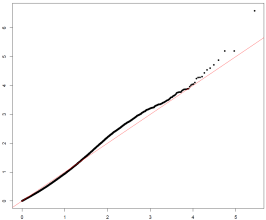

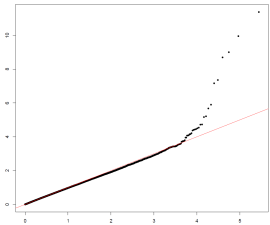

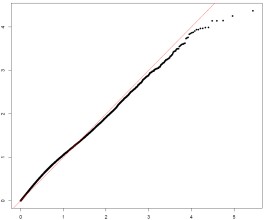

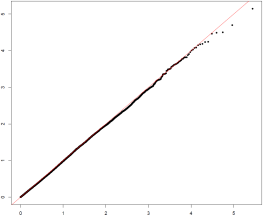

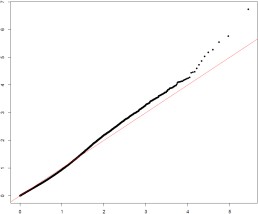


Chromosome Chromosome

**Figure S6.** mGWAS analysis. Selected Manhattan plots and QQ plots show association with the metabolites under well-watered (WW) and drought-stress (DS) conditions in three harvesti ng time points (19, 24, 45 DASt). The black horizontal line in the Manhattan plots represents the significance threshold of P-value = 1e-5).
